# Wildlife health investigations: needs, challenges and recommendations

**DOI:** 10.1186/1746-6148-9-223

**Published:** 2013-11-04

**Authors:** Marie-Pierre Ryser-Degiorgis

**Affiliations:** 1 Centre for Fish and Wildlife Health, Vetsuisse Faculty, University of Bern, Postfach 8466, CH-3001 Bern, Switzerland

**Keywords:** Collaboration, Definitions, Harmonization, Health, Impediments, Surveillance, Strategies, Risk factors, Tools, Wildlife

## Abstract

In a fast changing world with growing concerns about biodiversity loss and an increasing number of animal and human diseases emerging from wildlife, the need for effective wildlife health investigations including both surveillance and research is now widely recognized. However, procedures applicable to and knowledge acquired from studies related to domestic animal and human health can be on partly extrapolated to wildlife. This article identifies requirements and challenges inherent in wildlife health investigations, reviews important definitions and novel health investigation methods, and proposes tools and strategies for effective wildlife health surveillance programs. Impediments to wildlife health investigations are largely related to zoological, behavioral and ecological characteristics of wildlife populations and to limited access to investigation materials. These concerns should not be viewed as insurmountable but it is imperative that they are considered in study design, data analysis and result interpretation. It is particularly crucial to remember that health surveillance does not begin in the laboratory but in the fields. In this context, participatory approaches and mutual respect are essential. Furthermore, interdisciplinarity and open minds are necessary because a wide range of tools and knowledge from different fields need to be integrated in wildlife health surveillance and research. The identification of factors contributing to disease emergence requires the comparison of health and ecological data over time and among geographical regions. Finally, there is a need for the development and validation of diagnostic tests for wildlife species and for data on free-ranging population densities. Training of health professionals in wildlife diseases should also be improved. Overall, the article particularly emphasizes five needs of wildlife health investigations: communication and collaboration; use of synergies and triangulation approaches; investments for the long term; systematic collection of metadata; and harmonization of definitions and methods.

## Introduction

Growing human population, globalization, climate change and a number of ecological perturbations have resulted in an increasing number of emerging diseases. Given this context, the role of wildlife in human and domestic animal disease emergence has become widely recognized as a factor we can no longer afford to ignore. Thus, wildlife health surveillance has become an integral component in the identification and management of potential threats to human and animal health [1-4]. The objectives, concepts and methodology of investigations into wildlife health are similar to those of domestic animal health surveillance. But given the zoological, behavioral and ecological characteristics of wildlife populations, there are also some substantial differences that need to be taken into account when planning for, implementing and interpreting data from investigations into wildlife health. It requires a new set of definitions, methods and procedures, taking into account the unique conditions under which wildlife health surveillance is carried out [5]. Laboratories still tend to rely on domestic animal surveillance approaches in their investigations into wildlife pathogens. It is important to stress that although the growing interest in wildlife health is definitely a positive trend, this reliance bears a substantial risk of error at various levels ranging from initial study design all the way up to result interpretation.

This article reviews the factors to consider when investigating the health of free-ranging wildlife, including the challenges and constraints inherent in such investigations, with a particular focus on surveillance. The aims are to provide: (1) revised definitions of old concepts; (2) an update on the importance of influencing factors on wildlife health investigations; (3) an overview of tools available to wildlife health investigators.

### Health, disease and pathogen: modern definitions

The World Health Organization (WHO) has defined health as a “state of complete physical, mental and social well-being and not merely the absence of disease or infirmity” [6], which implies that most humans are unhealthy most of the time. In veterinary medicine, health has been defined as “a state of physical and psychological well-being and of productivity, including reproduction” and “health indices” refer to easily observed parameters that can be used as a guide to the animal’s or group’s state of health (e.g., food intake, fecal output, body weight) [7]. It has recently been proposed to redefine health as “the ability to adapt and to self-manage” [8,9], i.e., an organism is healthy if it is capable of maintaining physiological homeostasis through changing circumstances. If it is unable to mount a protective response, reducing the potential for harm and restoring an (adapted) equilibrium, damage remains and may result in illness [9]. Accordingly, measuring health is challenging and requires tools for assessing an individual’s capacity to cope and to adapt. It is also important to differentiate between the health status of individuals and that of populations [9].

Population health has emerged as an important discipline. In veterinary medicine it is particularly useful in the assessment of production animal health and wildlife health. Population health is concerned with the definition and measurement of health outcomes (the dependent variables) and the roles of determinants (the independent variables). The term “health outcome” is preferred over “health status”, because the latter refers to health at a point in time rather than to health over a period of years [10]. A hallmark of the field of population health is significant attention to the multiple determinants of health outcomes and their interactions, i.e., examining: systematic differences in outcomes across populations; the complexity of interactions among determinants; the biological pathways linking determinants to population health outcomes; the influence of different determinants over time and throughout the life cycle. While specific investigations into a single determinant, outcome measure, or policy intervention are relevant and may even be critical in some cases, they must be recognized as being only part of a more complex picture [10].

In accordance with the new definition of health [9], a disease is a non-balanced perturbation of one or more body function(s), including responses to infectious and non-infectious agents [11]. Disease can affect individual hosts by reducing growth rates or fecundity, increasing metabolic requirements, or changing patterns of behavior. It may ultimately cause death [12].

While many organisms have the potential to cause disease, infections usually have little detrimental effect on the host. Disease occurs if the delicate balance between hosts and parasites is upset, for example when the parasites become too numerous or when the immunological capability of the host is impaired [12,13]. Infection may also have sublethal effects that indirectly enhance mortality rates by for example, increasing the susceptibility of the infected host to predation [12].

A pathogen is usually defined as a microorganism that causes, or can cause, disease in a host [14]. Virulence and pathogenicity both refer to the ability of a pathogen to cause disease. However, while virulence is a continuous variable defined by the amount of damage that is caused, pathogenicity refers to the quality of disease induction and is a discontinuous variable (yes or no) referring to the capacity of a microbe to cause damage in a (susceptible) host [14]. It is not possible to draw a clear and unequivocal distinction between pathogens and non-pathogens. Properties conferring pathogenicity depend as much on the host as they do on the microorganism. They may be influenced by multiple factors such as environmental stress, pollutants and other microorganisms [15-17]. Such alterations can create conditions in which the host becomes vulnerable to microbes which were previously non-pathogenic. When the immune response of the host to a microbe is insufficient, microbial factors can cause damage; when microbial factors fail to stimulate the immune system, the microbe can disseminate and cause disease; and when the immune response to a microbe is too exuberant, it can be the immune response itself that is responsible for the pathology. In other words, pathogenicity can be due to the immune response to the pathogen rather than to the pathogen itself [14]. Consequently, attempts to classify micro- or macroparasites as pathogens, non-pathogens, opportunists, commensals and so forth are misguided because they attribute a property to the parasite that is instead a function of the host, the parasite and their interaction [14].

### Wildlife health investigations: data sources and factors to consider

#### Health surveillance: objectives and methodology

The World Organisation for Animal Health (Office International des Epizooties, OIE) defines surveillance (or epidemio-surveillance) as the on-going recording of diseases in animal populations with a view to disease management [5]. In short, surveillance means “information for action”. Its purpose is to support effective decision-making, including policy and priority setting, response to outbreaks and approval of trade movements. The outputs generated by wildlife surveillance systems can include the detection of new disease events, the demonstration of freedom from specific diseases or infections, or identification of the level and distribution of diseases endemically present in a population [18,19]. The first integral activities of disease surveillance are detection of disease or pathogens. Further activities include information management, i.e. analysis of the collected data and use of the surveillance information for decision-making and policy formulation [20]. For these two last aspects, it is essential to record and store data, e.g., to continuously conduct a database and to communicate important results such as disease emergence to the competent managers and authorities. Indeed, a key aspect of health surveillance is the early detection of outbreaks or what we call early warning systems.

Scanning surveillance (also called general or passive surveillance) refers to the recording of cases as they occur and are submitted for investigation. It usually corresponds to clinical surveillance aimed at detecting dead or visibly sick animals (followed by diagnosis or precise disease identification), i.e., identifying disease events in which wild animals are the victims [4,20]. Scanning surveillance is typically performed by investigation of animal carcasses or tissues from dead animals. The accurate identification of a mortality event requires a thorough pathological examination carried out by a wildlife specialist following standardized procedures and complemented by further laboratory analyses [5]. However, in the case of important pathogens such as zoonotic agents or those with economic importance for which wildlife hosts may act as healthy carriers, surveillance cannot be based on the collection of clinical data such as morbidity or mortality [5].

Targeted surveillance (formerly called active surveillance) is carried out when dead or living animals are proactively sampled specifically for the purpose of investigating them to detect a selected disease or pathogen, whether or not the infected or exposed wild animals are sick [5]. These investigations may focus on populations of apparently healthy animals [4,20]. Traditionally, targeted surveillance of wildlife has relied on a cross-sectional study design because this only requires a point sample (single capture or sample collection). Collected data most often relate to the identification of risk factors associated with disease, pathogen, or antibody prevalence. It is therefore essential to aim at a sample size that can provide reliable prevalence estimates and statistical comparison and to incorporate relevant biological, spatial and temporal variables [21].

**Figure 1:**
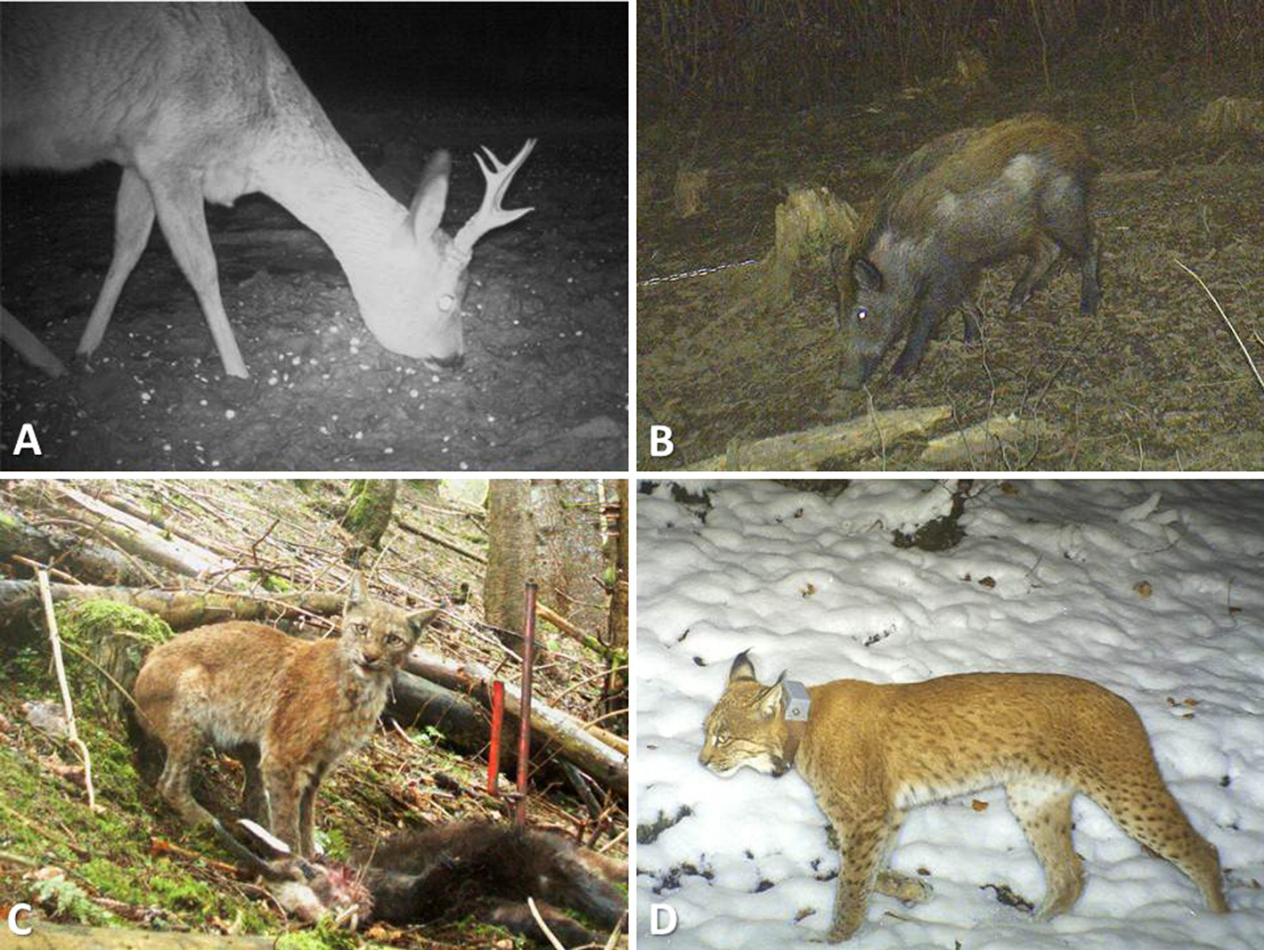
**Detection of diseased animals by camera traps set by hunters and game**-**wardens. A**. Marked enlargement of the lower jaw in a roe deer (Picture: Martin Wyler). **B**. First detection of sarcoptic mange in a wild boar in Switzerland (Picture: Alex Hofer). Notice the extended alopecia on the ventral parts of the body. **C**. Eurasian lynx affected by sarcoptic mange (Picture: Pierre Jordan). The emaciation and shaggy fur are obvious. This animal was subsequently captured, marked and treated. **D**. Same lynx as on picture **C**, six months after treatment (Picture: Pierre Jordan).

Although collection and sampling of animal carcasses represent a major data source for wildlife health surveillance, there are a number of alternative methods for accessing health information. Data from slaughtered farmed wildlife such as deer of from domestic animals potentially exposed to wildlife pathogens provide evidence of pathology as well as samples of blood and parasites [18,22-24]. Appropriate diagnostics (such as standardized health screening, ancillary diagnostic tests and thorough postmortem examinations) on diseased wild animals submitted to wildlife rehabilitation centers can enhance surveillance efforts [21,25-27]. Clinical examinations can be performed within the context of wildlife captures [28,29] and are an essential component of translocation programs due to the substantial health risks involved [30]. Photo-trapping can deliver valuable information about diseases with typical external lesions such as sarcoptic mange [31] (Figure 1). Infrared thermal imaging has been tested as a potential tool for the tele-diagnosis of sarcoptic mange in the Spanish ibex (*Capra pyrenaica*) [32]. Non-invasive samples, such as feces, hair and feathers may be suitable for the detection of pathogens or toxic compounds. For example, in a study on endoparasites in Alpine ibex (*Capra ibex ibex*), the number of feces samples collected from captured animals was insufficient for a comprehensive study and was substantially increased by collecting feces from the ground [29]. Finally, questionnaire surveys and interviews with wildlife managers, hunters and others working in the field can provide valuable information on disease occurrence.

Participatory approaches to surveillance have indeed been recently proposed as an interesting complement to more structured and quantitative methods [19]. It is an important principle of participatory epidemiology that stakeholders such as animal owners (or, in the case of wildlife, hunters or game-wardens) have valuable technical knowledge essential to understanding epidemiological scenarios. Obtained data can be validated by triangulation when information from diverse sources is collected. This method has been successfully applied to the surveillance of sarcoptic mange, a disease characterized by typical macroscopic skin lesions [33,34], and to document an epidemic of an alopecic syndrome associated with deer ked (*Lipoptena cervi*) infestation in Norwegian moose (*Alces alces*) [35]. Participatory approaches and triangulation have also been used to record interspecific interactions that may enable pathogen transmission [24,36,37].

Cutler and collaborators suggested that syndromic approaches might be a better way of disease detection than searching for specific pathogens [38]. Syndromic surveillance is meant as an early warning system aimed at identifying case clusters before diagnoses can be confirmed and reported to the relevant health agencies, in order to mobilize a rapid response [39]. A syndrome is a collection of frequently associated clinical signs putatively linked to a given etiology or to given risk factors. Syndromes usually refer to incompletely defined diseases. Thus, syndromic surveillance uses the clinical, pathological or epidemiologic characteristics of disease occurrences to assess whether these are linked, instead of relying on the detection of etiologic agents or well-defined disease pictures. The number of cases with defined syndromes can be tracked in space or time [4,5]. Macroscopic findings were indeed shown to be valuable for identifying distinct pathological profiles among collected wild animal carcasses. Besides the potential usefulness for early outbreak detection, this classification system represents a tool for retrospective investigations [40]. In a study on roe deer (*Capreolus capreolus capreolus*) mortality in Switzerland, classifying data according to syndromes has proven useful for describing morbidity and morbidity causes, including analyses of cases of unclear etiology [41].

Risk assessment should guide surveillance [42], in particular targeted surveillance (long-term data collection) or surveys (“snapshot” information). Risk-based investigations focus on areas where the probability of occurrence or the seriousness of the consequence for the target population is expected to be highest [5] and provide cost-effective, early detection of disease introductions or emerging health problems [19,21]. For example, targeted investigations on bovine tuberculosis in Swiss wildlife focused on areas along the border of the country, based on the knowledge that Switzerland is officially free of tuberculosis while the number of cases in wildlife has been increasing in all neighboring countries [43].

Adaptive surveillance corresponds to a cost-efficient, situation-based strategy for the surveillance of contagious diseases associated with noticeable morbidity or mortality in wildlife [44]. Investigations into the efficacy of using different information sources for detecting disease occurrence showed that analysis of indicator animals (i.e., animals found dead or presenting disease signs) is more efficient than analysis of animals expected to be healthy (such as hunted animals). This observation led to the recommendation of focusing surveillance efforts on indicator animals when a disease of concern appears to be absent in a wildlife population. Enhanced surveillance efforts, including systematic sampling of apparently healthy animals for prevalence estimation, should only be implemented when cases have been detected. Towards the end of an outbreak, the aim of disease surveillance should shift back to case detection [44]. This approach has been applied for rabies in Switzerland, where surveillance efforts progressively evolved towards clinical surveillance after rabies was eradicated [45].

#### Sample selection and diagnostic tools

Method selection for wildlife health surveillance should ensure repeatability and data quality. It is important to define which parameters to target (e.g., lesion, pathogen, antibody). Selection will depend on factors such as the host species, expected sample size, logistic constraints, cost of laboratory analyses and specificity and sensitivity of diagnostic tests [46]. A pathological examination is required to determine the cause of death or disease of an animal. This examination identifies tissue lesions and paves the way for appropriate additional investigations, such as bacteriological or parasitological examination. Also, the presence of lesions must be demonstrably associated with a given pathogen to prove that an etiological link exists between pathogen and death. Detection of exposure to pathogens is generally achieved by laboratory analyses such as agent isolation, PCR testing or serology [5]. To spare costs, it may be advisable to pool samples for analysis [37,46,47]. Non-infectious diseases may require toxicologic or genetic investigations. Toxicology is a discipline of major importance in wildlife, as both intentional poisoning and accidental intoxications frequently occur [48]. Illegal killing of wildlife is a widespread problem worldwide, and forensic approaches may be necessary when determining the cause of death in wild animals.

#### Molecular epidemiology

More studies should be performed on the pathogen strains circulating among wild animals and to compare them to strains of domestic livestock and humans. In many cases, researchers ignore whether or not strains circulating among domestic and wild populations are similar. As a consequence, epidemiological cycles of infectious diseases are not well assessed in any of the populations of concern [11]. In the case of infectious keratoconjunctivitis caused by *Mycoplasma conjunctivae* in Alpine ruminants, molecular studies revealed the simultaneous occurrence of the same strain of *M*. *conjunctivae* in different hosts of the same region, supporting the hypothesis of interspecific transmission [49]. Strains of *M*. *conjunctivae* identical to each other were also identified both in healthy and diseased hosts, suggesting that factors other than the strain of mycoplasma determine the outcome of an infection [50,51]. In contrast, comparison of *Brucella suis* from wild boar (*Sus scrofa*) and domestic pigs from the same geographical area presented differences that did not support the assumption that wild boar were the source of the outbreak in pigs [24,52].

#### Historic information and baseline data

Data and samples must be stored in a way that enables subsequent use for retrospective epidemiological analyses [4]. Baseline data and sample archives are needed: to establish the distribution of pathogens and appreciate their effects and epidemiology [53]; to assess perturbation over time and to understand the impact of climate and environmental change on complex systems of hosts and pathogens [42,53,54]; for early detection and implementation of control measures [55]; to determine whether or not the emerging character of a disease is due to the introduction of infected animals (pathogen pollution) [56]; and to assess the existence of a causal association between pathogen and disease [29]. For example, a survey on archived samples revealed that *Batrachochytrium dendrobatidis*, the causing agent of chytridiomycosis in amphibians, is more widely distributed in Europe than previously thought. This result was surprising considering that die-offs have only been infrequently reported from Europe [57]. As laboratory tools are continuously being developed, the access to archived material opens doors for new discoveries and allows us to learn from the past.

Besides the use of sample archives for retrospective studies, it may be necessary to accumulate samples gathered during several years until a required sample size is achieved [46]. This is especially true when access to samples is limited, as is the case in small and strictly protected animal populations. Thus, a study on *Trichinella* in Swiss carnivores included 1289 hunted red foxes (*Vulpes vulpes*) sampled within less than two years, while data from 55 Eurasian lynx (*Lynx lynx*) originated from necropsy samples taken over nine years [58].

#### Identification of risk factors

The identification of risk factors is an important goal of wildlife health research and surveillance [21]. Wildlife diseases may be indirectly managed by modifying population risk factors that enable the persistence of infection [59]. In the case of emerging infectious diseases, knowledge of risk factors is considered to be particularly important, as such data represent a tool for targeted surveillance and prevention such as improving decisions on land-use policies [46]. The same disease at the interface of the same two species may behave differently in different areas or situations, due to different climatic environments; different management of one or both species; shared food, water and mineral resources; population densities; predation variables; concurrent diseases, and so forth [21,60]. Therefore, sampling stratification is essential [46], and similar studies should be carried out in different regions with well characterized disease patterns. This will allow comparisons to identify risk factors for disease occurrence [42].

Prevalence of infection and disease susceptibility may vary among sexes and age classes. Just to give a few examples: endoparasite infestation is more frequent in male than in female Alpine ibex [29]; prevalence of antibodies to *Toxoplasma gondii* increases with age in Eurasian lynx [61]; and age- and sex-related variations have been noted in the epidemiology of Chronic Wasting Disease in mule deer (*Odocoileus hemionus*) [62].

The same infectious agent may have different pathogenic effects in different host species. First, some agents are truly species-specific. Second, susceptibility may vary among susceptible hosts. For example, only mammals are susceptible to rabies. Among carnivore species, canids, mephitids, procyonids and viverids are highly susceptible, while felids and mustelids show a lower susceptibility or receptivity [63]. Similarly, *M. conjunctivae* generally causes more severe disease in Alpine chamois (*Rupicapra rupicapra rupicapra*) than in Alpine ibex, and different strains of *M*. *conjunctivae* may or may not cause disease in either species [49-51]. Furthermore, species-specific social behavior influences the frequency and intensity of intraspecific contacts and consequently the transmission rate of infectious agents, the case incidence and disease spread among a host population [13,60]. The probability of contacts and risk of epidemic spread is therefore higher for social or gregarious animals than for solitary species. For example, while sarcoptic mange and distemper rapidly spread among red fox populations, cases in more solitary species like the Eurasian lynx remain isolated [28,64]. Gregarious lifestyle and inter-group movements have been shown to contribute to tuberculosis maintenance in some species despite low overall prevalence in the population [43]. Disease spread may also vary among sexes depending on the social organization of the species. In an epidemic of infectious keratoconjunctivitis in Alpine chamois in the Swiss Alps, numerous females and juveniles died while solitary adult males remained largely unaffected; after the mating season, the number of affected males dramatically increased [65]. Finally, interspecific interactions need to be considered when assessing the epidemiological role of a host species. Among other things, it is necessary to assess the real status of livestock to determine the epidemiological role of a wild species in domestic animal disease [11,46]. For example, investigations on bluetongue, bovine viral diarrhea virus and other abortive agents in wild ruminants from Switzerland suggest that wildlife is not a reservoir but rather an occasional spillover host for these pathogens, as infections of wild ruminants is only sporadic while domestic ruminants display significantly higher prevalences of infection [37,47,66]. Interspecific interactions can also influence disease dynamics in other ways. Thus, marked advances of the epidemic front of sarcoptic mange in Sweden were associated with new vole cycles, possibly because years with high vole numbers (i.e., high prey numbers for foxes) result in high reproduction and juvenile fox survival rates and thus in enhanced fox dispersal [67].

The possibility of temporal and spatial variation always needs to be considered in both study design and data interpretation [21,68]. Time is a quantitative scale against which all other aspects of disease can and should be measured [69]. The season of sampling should be considered whenever assessing the risk of infection by pathogens [70] or using wildlife as an accumulative bioindicator of environmental pollution [11]. Pathogen transmission is affected by climatic conditions (survival of the pathogen in the environment, activity of arthropod vectors, geographical distribution of host species) [18] as well as by intraspecific contacts (e.g., mating season) [65] and interspecific interactions (e.g., presence of livestock on alpine pastures during the summer grazing season) [49,71]. Severe weather events can affect host immunity and nutrition if they interrupt forage availability and feeding patterns [18,70], as well as the abundance of arthropod vectors [13]. Seasonal changes in hormonal activities may also influence host susceptibility [72]. Because seasonal changes can be related either to climatic conditions or to physiological and behavioral patterns, it has been proposed that seasons be specified both as calendar seasons and as species-specific biologic periods [51,73]. An example of climate-related seasonal disease pattern is the observed increased prevalence of sarcoptic mange in Iberian ibex (*Capra pyrenaica*) in the Sierra Nevada in winter and spring months, probably because low temperature and high relative humidity are favorable to mange mites [74].

Migratory behavior can strongly influence the transmission and prevalence of pathogens in a given species and geographical area [21]. Migratory behavior is strongly linked to seasonal changes and typically allows for contacts with other hosts and thus for pathogen spread and acquisition. For example, vertical short-distance migration in red deer (*Cervus elaphus elaphus*) in the Swiss Alps and food shortage in winter enhances the risk of contacts between red deer and cattle during the cold season. When red deer move to lower altitudes and share food resources with domestic livestock, a situation known as a risk factor for pathogen transmission arises [37]. Prevalence of low pathogenic avian influenza virus in ducks in the northern hemisphere is highest during late summer and early fall (pre-migration staging) and declines during winter [68]. More importantly, horizontal long-distance bird migration has most likely played a major role in the spread of highly pathogenic H5N1 avian influenza in Europe [75]. Finally, biological dispersal (animal movement away from an existing population or from the parent organisms) is known to contribute to pathogen spread. For example, red fox dispersal is believed to favor the propagation of sarcoptic mange [67].

The study of spatial distribution is especially important when the targeted disease or infection is highly clustered [21]. Spatial epidemiology also enables to investigate relationships between the environment and the presence of disease, and to predict disease spread [76]. Natural barriers such as rivers and mountain ranges can temporarily block disease spread, as has been observed for infectious keratoconjunctivitis in Alpine chamois and for rabies in red fox in Switzerland [65,77]. Spatial representation of pathogen exposure in different species can also contribute to the understanding of epidemiological roles. Thus, investigations in Switzerland suggest that infections of wild ruminants with bluetongue virus are associated with foci of infection in domestic ruminants [47]. Similarly, exposure of wild ruminants to the viral bovine diarrhea virus seems to be related to their presence on summer Alpine pastures where interactions with cattle regularly occur [37]. Differences in human population density have been shown to be associated with significantly different exposure to *Toxoplasma gondii* and Orthopoxvirus in Swedish Eurasian lynx. This could relate to the concomitant presence of domestic cats in the area [61,73].

Differences in food availability among geographical areas can be expected to influence host density and thus the pattern of pathogen spread. For example, sarcoptic mange has had a stronger impact on red fox populations in boreal forests than in mixed agriculture-forest areas of Sweden. This is presumably because groups of foxes are larger and live in smaller territories in highly productive regions, and because the more patchy distribution of food and shelters favor disease spread [78]. Environmental characteristics such as temperature and humidity can impact pathogen survival. Thus, foxes are more often infected with *Echinococcus multilocularis* in humid than dry environments [79]. Pathogen and disease distribution can also vary with altitude (due to variations in the distribution of susceptible hosts and in the occurrence of arthropod vectors) and with sun exposure (influencing climatic conditions and thus pathogen persistence in the environment) [18]. The results of recent investigations on infectious keratoconjunctivitis in wild Caprinae have suggested a role of altitude (or associated environmental conditions such as UV-light, temperature, humidity) in pathogenesis of eye lesions due to *Mycoplasma conjunctivae*[80]. Also, soil composition, vegetation and stagnating water can influence the occurrence of pathogens and their vectors or the presence of toxic compounds [18]. Overall, natural and anthropogenic barriers and spatial distribution of wild and domestic hosts need to be taken into consideration when defining epidemiological or sampling units [37,50,66,81].

Recording disease-related information in a format compatible with the Geographic Information Systems (GIS) enables analyses on the potential influence of factors such as climate, forest type, stream location, relief and human demography on disease and pathogen occurrence [18]. The system can, for example, easily derive the altitude of sampling locations from the geographical coordinates [47,80].

#### Research, experiments and modeling

Descriptive epidemiology deals with the occurrence and extent of a disease as well as the frequency and distribution of risk factors in populations (what, who, where and when; e.g., [65,82,83]). However, analytical and experimental approaches are also needed to study causal relationships (how and why). Controlled experiments allow for a better understanding of pathogenesis, contribute to the development and validation of diagnostic methods and to targeted management recommendations [21,56]. Experimental studies should ideally combine lab experiments (e.g., experimental infections) with field experiments (e.g., testing hypotheses regarding the effect of host aggregation and density on disease prevalence or pathogen persistence). Furthermore, mathematical modeling may help to identify knowledge gaps and design experimental research [56]. Models can also contribute to the understanding of disease dynamics and the impact of diseases on populations [84,85]. However, it is important to remain cautious in basing models on extrapolations of information about other species, as this can lead to erroneous conclusions [86].

#### Method harmonization

Coordination between wildlife monitoring programs and standardization of both diagnostic protocols and protocols for estimation of population abundance among the different regions and countries are needed. Such coordination will allow a global and harmonized evaluation of disease status and associated risk factors [11,56]. As an example, a recent attempt to compare worldwide prevalences of bovine tuberculosis and host population densities (considered as an important risk factor for disease maintenance) revealed that such a comparison is nearly impossible due to the diversity of diagnostic and counting methods applied in the various geographical areas [43]. Important steps towards the harmonization of methods have recently been initiated. Examples are the publication of the Training Manual on Wildlife Diseases and Surveillance by the OIE [87], the publication of Diagnosis Cards by the EWDA (http://www.ewda.org) and the initiation of international research projects such as WildTech (http://www.wildtechproject.com) and APHAEA (http://www.aphaea.eu).

#### Multidisciplinary approaches

There is a need to adopt a multidisciplinary and integrative approach in investigations on wildlife health, including both research and surveillance [1,53]. Early warning and response systems for emerging zoonoses require effective cross-jurisdictional and interdisciplinary collaboration [21]. Also, the integration of perspectives and expertise from diverse branches of the natural and health sciences such as ecology, zoology, veterinary medicine, human medicine, microbiology and pathology, as well as from the social sciences, into studies of emerging human and domestic animal disease is essential to understanding the role of environmental changes in disease emergence [1,42]. Veterinarians and other health professionals must become integral parts of the teams dealing with wildlife management and research [88]. In the frame of field projects, veterinarians ought to participate fully in project planning and realization and should not only become involved when emergencies occur such as injured animals or critical wildlife immobilization events [30,59]. Similarly, veterinarians must collaborate with experts in other disciplines when addressing questions related to wildlife health. In particular, cooperation with field ecologists and wildlife managers with a good knowledge of the local host populations is invaluable.

#### Human dimension: communication and politics

As the importance of wildlife health is increasingly recognized, the need for high standards is more and more emphasized by the scientific community. In contrast, the human dimension of wildlife investigations is rarely addressed. This is rather surprising as the collaboration with field professionals, local managers and the general public, is usually essential to accessing information on wildlife health. Nevertheless, there has been a recent recommendation for taking the country’s historical and cultural context into account in the frame of wildlife sampling [4]. The increasing interest in participatory epidemiology also underlines the need for a customer-oriented approach. Pro-active work in the field and the delivery of surveillance outputs that are meaningful to stakeholders are required, because motivation to carry out effective disease surveillance will decrease in the absence of adequate feedback [19]. Participatory approaches, translated into the field of wildlife disease investigation, mean improved communication between hunters or wildlife managers and animal health services. Presence of researchers in the field (e.g. participation in sample collection, courses) combined with providing regular feedback to field partners positively influences the access to general information on disease occurrence and facilitates sample collection [89].

It is essential to pay careful attention to the knowledge transfer and academic-practice partnerships [10]. Active education campaigns must be based on current science and avoid reacting defensively to rumor and misinformation [86]. The public must come to understand that the disruption of complex relationships within the ecosystem may have disastrous unforeseen consequences for human health. Patz and collaborators have gone so far as to recommend selecting research questions collaboratively with the local community and decision makers and fully integrating research findings into the social, economic and political dialogue, both globally and locally [42]. What this boils down to is quite simple: adequately informing the public in order to generate political will for effective change.

Finally, as free-ranging wildlife does not respect political borders, interregional and international communication is essential for early disease detection and timely implementation of preventive measures. For example, classical swine fever has emerged in southern Switzerland due to movements of free-ranging wild boar coming from Italy [90], and the spread of H5N1 avian influenza in Europe has been largely due to migratory flows of wild birds [75]. Overall, networking is a key factor in building and sustaining surveillance and response capacity against existing and emerging diseases [21].

#### Training and education

It is critical to build up specialized programs and to avoid the temptation to simply transfer the methodology usually applied to domestic animals to wildlife [59]. There is currently an obvious need for professional education in wildlife health both at the undergraduate and postgraduate level [56,59,88]. However, while curricula of veterinary students, at least in Europe, continue to devote only a small number of hours to wildlife diseases, an increasing number of postgraduate education programs are being offered in wild and zoo animal medicine [91]. Most recently, the European College of Zoological Medicine (ECZM) has developed the specialty “Wildlife Population Health” and is currently setting up the corresponding residency program (http://www.eczm.eu).

Since there is only a limited number of appropriately trained veterinarians and positions available, it is essential that those actively involved in these fields train and impart knowledge to non-veterinarians [88]. There is a general need to initiate regional training for capacity building to improve disease surveillance in wildlife [70]. Among others, many biologists are not familiar with wildlife pathogens and health disorders [4]. To contribute to filling these gaps, the OIE has published manuals such as “Quarantine and Health Screening Protocols for Wildlife prior to Translocation and Release into the Wild” [92], “Post-mortem Procedures for Wildlife Veterinarians and Field Biologists” [93] and the more recent “Training Manual on Wildlife Diseases and Surveillance” [87].

#### Sharing existing information

In earlier times, knowledge acquired from wildlife health surveillance was published – at most – in the local language in local journals. Publication of scientific articles in international revues is now increasingly recognized as a necessary means of communication for the worldwide exchange of information, and the possibilities for distributing and accessing new data are becoming more numerous, easier and cheaper.

Scientific institutions increasingly require scientists to fulfill expectations regarding the quantity of published articles and the mean impact factor of the selected journals. Funding attributed for future research may even depend on these factors. While this pressure encourages researchers to publish their data, it may discourage them from reporting information arising from small sample sizes, which is considered less valuable than results from more elaborate, larger studies. However, the difficulties inherent in wildlife health investigations (see below), the need for baseline data, the complete lack of reports in certain regions of the world or on secretive species, may render anecdotal information or negative results valuable for surveillance purposes. Also, it is important to keep in mind that publication is a means of communication for sharing experience and information, and not primarily a vehicle for prestige and recognition. Negative data are greatly underrepresented in the literature despite the fact that they can be extremely important to our understanding of diseases [21]. Among others, the absence of pathogen exposure detection provides necessary baseline data in the surveillance of emerging diseases. Similarly, the value of publishing failed experiments is underestimated. It is important that we benefit from each other mistakes and not continue to reinvent the wheel [94].

### Difficulties inherent to health investigations in wild populations

Surveillance and research on wildlife-related diseases are associated with a number of challenges which include not only the practicality of the case, sample and field data collection, but also the interpretation of field data and the validation of field observations through experimental studies [21]. A further difficulty associated with wildlife is its wide taxonomic diversity [4]. This requires knowledge of zoology (species identification) and of species-specific issues such as anatomy, pathology, disease susceptibility and ecology.

#### Access to investigation material

Wildlife case submissions are dependent on a complex of interrelated natural and decision-making outcomes [21]. Scanning surveillance requires the observation of clinically diseased or dead animals, reporting these observations, and the submission of carcasses or samples by the public and field professionals for analysis. Overall, only a very small portion of dead wildlife is both found and examined. Wild animals may inhabit remote areas and are often difficult to approach and examine; clinical expression may be brief [5] and survival behaviors often mask clinical signs of disease in wildlife [86]; capture and containment of moribund animals may not be feasible and represents additional steps to be surmounted in the submission chain [21]. Predation, scavenging and carcass decomposition may obscure the observation of dead animals and usually complicate the diagnostic process when carcasses are found [21,86]. Detection biases can arise from the cause of death. For example, a study on Eurasian lynx showed that infectious diseases are underrepresented in animals found by chance while mortality resulting from anthropogenic activities is overrepresented [95]. The use of dogs has been shown to be an efficient method for increasing detection of diseased or dead animals, including medium-sized to small mammals particularly difficult to detect, and thus of decreasing bias in the recovered study material [96,97].

**Figure 2:**
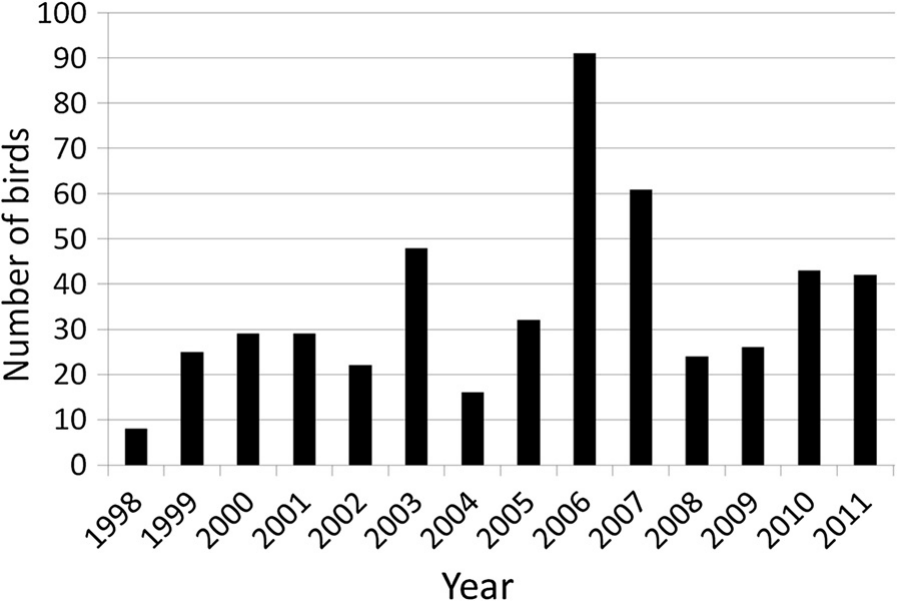
**Yearly numbers of bird carcasses submitted to the Centre for Fish and Wildlife Health, Switzerland.** The peak of submissions in 2006–2007 was associated with the avian influenza outbreak in Switzerland [101] and the recrudescence of cases in 2010 was partly due to an epidemic of salmonellosis in passerine birds [99].

Reporting efforts are strongly associated with personal interests, education and contacts with competent laboratories [69]. The perceived value of individual species (e.g., game vs. non-game) or perceived need for a submission (mass mortality vs. single case, awareness of pathogen spread) will largely influence the decision of whether or not to submit a carcass for pathological investigation [21]. Thus, after the first cases of H5N1 highly pathogenic avian influenza were discovered in Switzerland in 2006, a significant rise of bird carcass submission was noticed at the Centre for Fish and Wildlife Health (FIWI) in Bern although all potential influenza cases were sent to the reference laboratory at Zurich University (Figure 2). Similarly, the number of submitted red foxes and Eurasian badgers (*Meles meles*) dramatically increased during an epidemic of distemper in wild carnivores [64], including cases that had died of other diseases (FIWI archives, unpublished data). However, despite the listed impediments, scanning surveillance retains considerable potential value. It provides an ideal setting for disease discovery, in particular of emerging diseases of wildlife, as has been the case for chronic wasting disease and bovine tuberculosis in the United States [21], for babesiosis in Alpine chamois and salmonellosis in passerine birds in Switzerland [98,99] or for avian pox in British tit species [100].

Targeted surveillance and research in wildlife require proactive sample collection. To guarantee a certain level of detection and accuracy, the number of samples needed is a function of the population size combined with the expected number of positive animals. When an expected prevalence is not known, sampling intensity should focus on finding at least one positive animal assuming a very low prevalence, which often requires a very large sample size [68]. This approach has been successfully used in studies on bluetongue virus and bovine viral diarrhea virus infections in wild ruminants in Switzerland [37,47]. Ideally, samples originate from sources that do not compromise animal health. However, capture options are often limited. Wildlife trapping requires a considerable know-how, is extremely demanding in both time and financial resources and may result in very low sample sizes [21,94]. For example, in Switzerland in the winter of 2011/2012, six months of “capture duty” with three different capture systems [102] and the contributions of numerous biologists, veterinarians, hunters and professional game wardens, resulted in the capture of four Eurasian lynx. Similarly, three capture seasons of 2–3 months each involving full-time presence of a capture team in the fields on a single study site resulted in samples from 39 ibex [29], while blood samples from 520 hunted ibex were obtained during the corresponding hunting seasons [66]. Additionally, legal constraints may prevent systematic sampling of a wild animal population [94].

Sample collection during hunting season generally ensures access to a large amount of samples but it depends on hunters’ compliance and hunting success. This method is also limited to specific periods of the year and geographical areas. Hunted animals are often selected based on nonrandom criteria such as antler development or on hunting plans and may not be representative of either the age or gender structure of the population. In addition, capture or killing of animals for specimen collection may result in the rapid dispersal of contagious animals [86]. Consequently, plans for general surveillance are often limited to opportunistic sampling of animals using whatever means are available to collect specimens for necropsy and laboratory tests [4]. Even in the frame of targeted investigations, sampling largely results in convenience sampling despite strong efforts towards representativity and stratification. Overall, sample collection of free ranging wildlife usually represents a compromise due to availability [21].

Sampling materials should be selected based on how easy they are to collect, transport and ship and on refrigeration requirements, due to sampling conditions in the field and due to the necessary involvement of non-veterinarians in most sampling campaigns. In the case of large sample sizes, sample volume and shipment costs also need to be considered [103-105]. Depending on the objectives of the study, samples such as swabs or body fluids may represent a suitable alternative to tissue collection and significantly increase hunters’ compliance. Filter papers seem to be a promising tool for antibody surveys, as they are an inexpensive sample media well suited for harsh environmental conditions [103]. Similarly, FTA technology (fast technology for analysis of nucleic acids, i.e., a paper-based system designed to fix and store nucleic acids directly from fresh tissues) represents an alternative method when field conditions limit the ability to properly store or ship traditional sample materials. Among others, the cards can inactivate infectious agents, which reduces the risk and restrictions associated with transporting samples [105]. When samples like blood or body fluids are not obtainable from carcasses, the use of fluids obtained from organ samples such as lung extract [106] or meat juice [107] may be considered.

#### Diagnostic limitations

Tissue and fluid degradation in carcasses that have remained in the environment for an extended period of time poses serious diagnostic problems. Autolysis greatly limits the interpretation of gross necropsy and histologic findings. Degradation also influences sensitivity of diagnostic tests. Furthermore, the use of often highly contaminated bloody fluids collected from carcasses may result in noninterpretable or unreliable test results. However, studies on red foxes and wild boar have demonstrated that hemolytic body fluids are suitable for serosurveys. Hemolysis may result in a loss of sensitivity, however it seems that storage time and temperature as well as thawing-freezing cycles influence the results more than hemolysis itself [47,106,108,109].

Optimum tissues or samples for pathogen detection may vary between species or between systems [21]. For example, nasal swabs have proved effective for detection of Aujeszky disease virus in domestic pigs but not in feral pigs [110]. Differences in viral loads among different hosts, the occurrence of cross-reacting antigens and sequence similarities among closely-related pathogens can also significantly affect the reliability of diagnostic test results. It is therefore strongly recommended to validate serological tests with experimental trials and, in the case of PCR-based diagnostics, to carry out amplicon identification through sequencing [21].

There is a need to develop diagnostic tests appropriate for wildlife species. Many tests designed for domestic mammal samples do not have the same levels of sensitivity and specificity when used in wild species [5,56]. Whereas tests aimed at directly detecting the pathogen usually give similar results in both domestic and wild animals, indirect tests such as ELISA – which are based on detecting the immune response of the host to the pathogen and thus depend on the recognition of specific proteins associated with that response – may not deliver reliable results [5]. Validation of diagnostic tests in wildlife is associated with a number of challenges such as the difficulty to obtain enough positive and negative controls, the lack of gold standards, the large number of animal species and limited financial resources. Nevertheless, efforts made to overcome these problems are increasing. For example: the EWDA Wildlife Health Surveillance Network has initiated the edition of Diagnosis Cards recommending diagnostic techniques appropriate for wildlife testing (http://www.ewda.org); the WildTech Project aims at developing new technologies for the improvement of wildlife health surveillance (http://www.wildtechproject.com); and the APHAEA project will propose harmonized methods for diagnostic investigations in wildlife (http://www.aphaea.eu).

New diagnostic tools may also contribute to overcoming difficulties related to sampling conditions and the limitations of traditional diagnostic tests. Loop-mediated isothermal amplification (LAMP) and RT-LAMP have several advantages over PCR and RT-PCR. They can be carried out at constant temperature in a single tube with conventional instruments; the reaction requires a shorter time; the result can be judged with the naked eye and without opening the tubes; and specificity is higher [111]. Genomic tools such as microarray-based analysis of pathogens are new technologies that overcome the technical limitations of current analytical methods (e.g., phylogenetic analysis, proteotyping) and are expected to contribute to the rapid identification of newly emerging disease agents [112].

#### Impediments to experimental studies

Experiments in wildlife are associated with a range of difficulties, including animal procurement, husbandry and housing requirements, artificial infection route or doses, costs related to extended studies and a number of unrecognized variables such as genetics or exposure history. Furthermore, important environmental factors relevant to the pathogenesis are not present and consequently experiment results may not reflect the field situation [21]. For example, an experimental infection could only reproduce mild signs of infectious keratoconjunctivitis in Alpine ibex. It was unclear whether this was related to the strain of *M*. *conjunctivae* selected for the experiment, or to unknown environmental factors playing an important role in disease pathogenesis [113]. It is therefore essential to cross-validate and question both experimental and field data [21].

#### Distinguishing disease from infection

Pathogen distribution and prevalence may be larger than disease distribution and prevalence. In a susceptible animal, exposure may lead to inapparent (or silent/subclinical) infection or to clinical infection with disease signs of variable severity and resulting in different outcomes including chronic illness, recovery, or death. Despite the absence of clinical signs, infected animals may shed the pathogen either continuously or intermittently. Such a carrier status may occur in inapparent infections (healthy carriers), during incubation (incubatory carriers), or during recovery (convalescent carriers) [114]. Considerable misunderstanding has arisen from using serosurveys for wildlife disease surveillance because the distinction between infection (past or current) and disease is not always recognized. This leads to the misconception that infectious diseases are widespread in free-ranging wild populations. When an infectious agent is endemic and the host has evolved in this given environment, disease is the exception, not the rule [13]. For example, the hemoparasite *Cytauxzoon felis* is widespread in healthy bobcats (*Lynx rufus*) and only rarely associated with clinical signs, but it generally causes fatal acute disease in domestic cats [115].

#### Linking molecular and descriptive epidemiology

Molecular epidemiology uses methods for characterizing DNA or protein amino acid sequences in conjunction with epidemiologic analyses to describe the distribution and determinants of disease in defined populations. It is an extremely valuable tool but conclusions about the origins and behavior of infectious agents denoted solely from molecular findings can be misleading [116]. To assess the epidemiological role of different hosts, it is essential to combine results from molecular analyses with field data, prevalence and risk factor studies.

#### Lack of basic knowledge about host species

A unique aspect and challenge to epidemiologic studies involving wildlife relates to the need to integrate the collection of both disease and basic biological data [21]. Technical difficulties often include the lack of baseline information on the disease and the population in question [29]. There is also a lack of basic knowledge (such as susceptibility, carrier status, transmission potential) concerning many diseases in wild species [86]. Importantly, there is often a lack of information on natural history, behavior, anatomy and physiology of wild species. Food habits, basic nutritional requirements, home range size, gross and microscopic appearance of normal tissues, concentration of blood constituents, expected parasite fauna and age at first reproduction may represent critical data for understanding a disease. Therefore, the study of disease in a wild species often must include a substantial investigation into the basic biology of the host species [69].

#### Inaccurate age classification

Age is often a critical variable in epidemiologic investigations but there is a lack of defined age criteria for many wildlife species; furthermore, even if available, these age criteria usually allow only gross categorization such as juvenile vs. adult, resulting in a lack of precision. The first limitation associated with age is related to exact age determination. Various methods have been described in fur animals [117]. Horn growth delivers accurate information in bovids while antler size in cervids is useless. Tooth wear provides inaccurate data of limited reliability in case of a sole macroscopic examination. Counting horn cementum annuli of tooth roots allows exact determination of age in many animals and is often applied on carnivores [73,118] but it is very expensive. This method is less appropriate in hunted game from which trophies are systematically collected. Furthermore, tooth extraction for the sole purpose of determining age is questionable in live animals.

The second limitation is related to the lack of meaningful and harmonized definitions of age classes. In particular when addressing questions related to disease transmission and intra- or interspecific interactions, it is important to consider the species-specific behavior of the host when defining age classes [50,51,73].

#### Missing population data

Population data needed in an epidemiologic study may include population size, density, age structure, sex ratio, recruitment and losses, home range size, habitat utilization, occurrence of sympatric species and migration behavior. Such information is often critical to understanding pathogen transmission and maintenance within wildlife populations [21]. Population size is also required to determine the sample size needed for prevalence estimation. Furthermore, health surveillance data have to be combined with the monitoring of wildlife abundance and the study of wildlife ecology to determine whether the emerging character of a disease is due to the introduction of infected animals (pathogen pollution) or to a change in the population dynamics of a host or vector [56].

Obtaining a census or even reliable estimate of the population is problematic for many wildlife populations [86]. Population data often are in the form of an index rather than of a true estimate. Such indices can be used to demonstrate trends but give no information on population numbers, densities, or spatial distribution [21]. Consequently, the proportion of cases in a sample can only be considered as an indication of the probability of infection or exposure to the pathogen [5]. The identification of population impacts due to disease, which represents another important goal of wildlife health investigations, is also particularly challenging [21]. Overall, there is an urgent need to develop improved methods for estimating animal abundance [5,43].

#### Difficulties in comparing data

In addition to the needs to harmonize methods of diagnostic testing, of estimating population abundance and of age classification, further factors may impede data analyses. The access to accurate information regarding the origin of the sampled wild animals can be difficult, as coordinates are not systematically submitted with samples. Also, points of capture and sampling are not necessarily representative of the living environment of animals, especially in species with extended home ranges [21,80]. This problem can be partly overcome by using geographical units for analyses, but data on different hosts are rarely available at the same spatial resolution and at a high enough resolution to allow meaningful inferences to be made [46]. Furthermore, there is often a lack of data concerning species interactions as well as the infection status in other species [11,37,46,66], and it is rarely possible to follow up on individual animals [37,51,69].

When analyzing surveillance data, interpretation must take into account the constancy and uniformity of surveillance pressure [4]. Both the intensity of surveillance efforts (related both to the program design and to the skills and personal interests of the involved personnel) and the method selection for surveillance (including diagnostic methods) may vary over time and limit the comparison of results. Furthermore, there are no long-term records of disease prevalence or baseline estimates of disease impacts on fitness for most wild populations [54]. Even if old records exist, they may be only available in paper form and difficult to understand or read; database software now facilitates information storage, however, multiple changes of software over time may render access to former digital reports impossible.

#### Political and financial restrictions

In addition to science-related difficulties, political and financial difficulties play a major role in the attempt to perform disease surveillance and management in wildlife. Overcoming these difficulties requires great efforts in interagency team building (biologists, animal health specialists, public health investigators, authorities) and involvement and education of the public [86].

## Conclusions

In a fast changing world with an increasing number of emerging diseases affecting wildlife, domestic animals and humans, the need and interest for effective wildlife health investigations including both surveillance and research, is now widely recognized. The lack of surveillance schemes is often mentioned as a cause of emerging diseases. In contrast, wildlife health surveillance produces knowledge that benefits at least three different agencies, namely animal health, public health and conservation [46]. However, methods applicable to and knowledge acquired from studies related to domestic animal health can only partly be extrapolated to wildlife.

In a former review, Boadella and collaborators formulated six recommendations for monitoring of wildlife diseases: to perform monitoring in relevant domestic animals and/or humans in addition to investigations in wildlife; to consider background information on wildlife population ecology; to select the appropriate wildlife hosts for monitoring; to select appropriate methods for diagnosis and time/space trend analysis; to define the target parameters for monitoring; and to establish a reasonable sampling effort and suitable sampling stratification [46]. The present review is in agreement with these recommendations but emphasizes five further needs of wildlife health investigations: (1) communication and collaboration (human dimension, networking and publication); (2) use of synergies and triangulation approaches; (3) investments for the long term; (4) systematic collection of metadata, i.e., information on the sampled animals such as age, sex and geographical origin; (5) harmonization of definitions and methods.

Participatory approaches, networking and transdisciplinary communication are key factors in efficient wildlife health surveillance. First, reports of unusual health events and the submission of carcasses largely depend on disease awareness, personal interests and the good will of the public and of field professionals. Second, targeted studies dealing with infectious pathogens generally require an effective collaboration with hunters, as the carcasses of hunted animals are an irreplaceable source of samples. In this context, direct human contacts are essential for a sustainable surveillance system. Close interactions with field partners and regular feedback should be an integral part of any project requiring wildlife samples. Sharing knowledge is a bilateral process in which all involved partners give and receive. However, as new technologies are developed, as software systems allow better data storage and as electronics facilitate rapid communication, the human dimension of wildlife health investigations tends to be neglected. Along similar lines, Nature recently quoted an epidemiologist as warning against the “mirage of technology” in surveillance; he emphasized that “the labs’ top priority should be building teams of local staff who are familiar with the region, its language and practices, such local knowledge being crucial to interpreting data” [119]. Nevertheless, when non-professional field partners are recruited for sampling campaigns, it is essential to provide them with adequate equipment and information to ensure personal safety.

Interdisciplinary collaboration is not only necessary for a comprehensive approach to wildlife-related health issues, it is also an enriching experience for everyone involved. But mutual respect is crucial for effective and productive cooperation. Open minds and curiosity for each other’s field of expertise are essential. Also, considering the difficulties in accessing wildlife samples, the challenges encountered in the frame of laboratory analyses and the value of population biology in interpreting results, the contributions of all parties should be equally acknowledged, from project planning to data publication.

Per definition, surveillance is carried out with the goal of providing data useful for developing management strategies. Knowledge obtained through surveillance efforts should be made available not only to local services but also to the international scientific community. The growing number of professional networks accessible via internet platforms and email groups provides the opportunity to exchange information rapidly and efficiently. Also, the number of published articles in scientific journals is exponentially increasing. Unfortunately, publications are partly promoted for prestige purposes. This attitude bears the risk of neglecting less impressive but useful data, such as negative results, baseline values or case reports. Furthermore, grants are generally allocated for new data collection but not for the analysis and publication of already existing data.

To be effective and comprehensive, a surveillance program should include various components investigating different aspects of health events which serve to complement one another, such as scanning and targeted surveillance approaches, outbreak investigation, archiving of biological samples, field and laboratory studies, predictive modeling and risk assessment [19,53]. Similarly, for targeted investigations a triangulation approach, such as the combination of antibody or pathogen surveys with pathological investigations, or of laboratory or field investigations with questionnaire enquiries, may be useful to access a satisfying amount of data and increase result reliability. Different methods may also act in synergy: presence in the fields for targeted sampling usually increases disease awareness in hunters and game wardens, who may subsequently submit more cases for scanning surveillance; feedback from the laboratory to the fields in the form of reports on investigated carcasses will encourage field partners to participate in future sampling campaigns; teaching efforts also contribute to the success of surveillance by increasing the interest of field partners for health issues; conversely, data arising from surveillance are useful for teaching purposes. Overall, synergies not only improve the efficiency of the system, they also permit saving resources.

Surveillance is an on-going process unlimited in time. For this process to be efficient, it is important to work with long-term goals in mind. Suboptimal communication with field partners in the framework of a single survey may compromise future studies and scanning surveillance efforts. Furthermore, sample and data archives not only allow retrospective investigations, they also contribute to saving resources and increasing sample sizes in future surveys.

Systematic collection of metadata during sampling campaigns is necessary because various environmental and individual factors can influence results obtained in the framework of surveys. Also, collection of wildlife specimens is often hampered by issues related to population management and access to the animals, resulting in a convenience sample. The distinction between risk factors for infection and sampling biases is crucial. Furthermore, definitions and methods – for fieldwork, laboratory analyses and data management – need to be standardized to allow for a global approach to wildlife health investigations.

**Figure 3:**
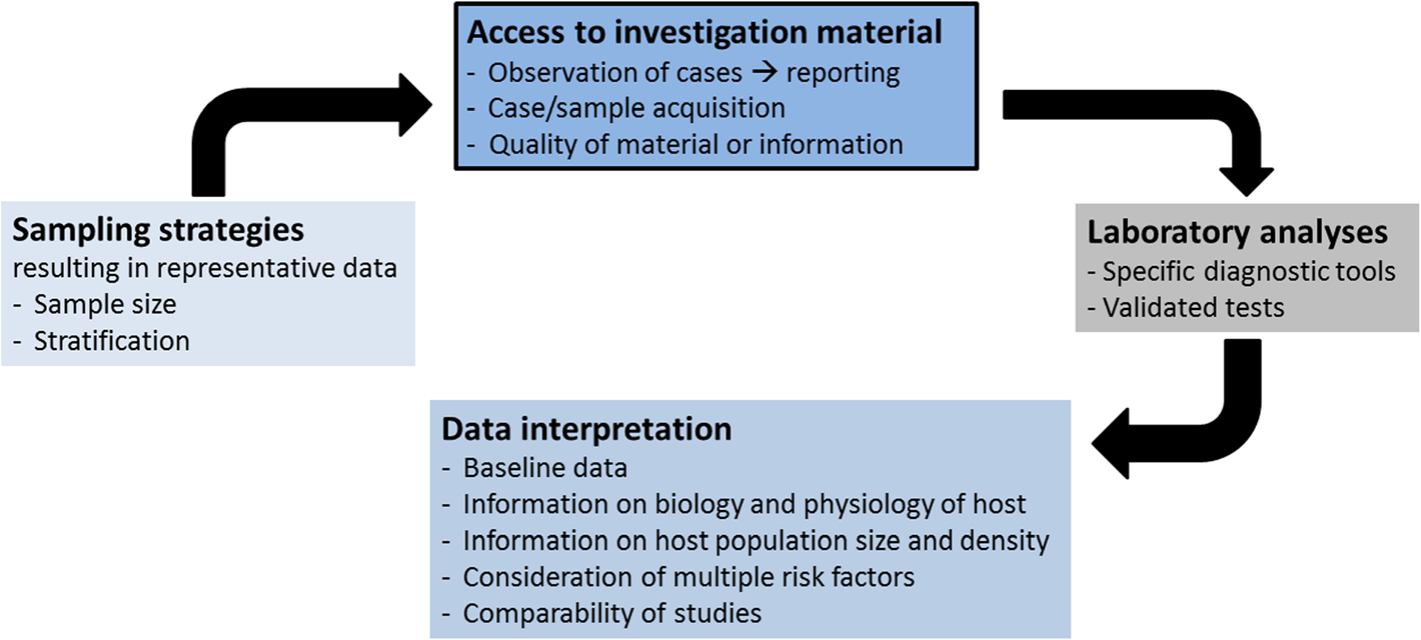
Challenges inherent in wildlife health investigations.

Overall, wildlife health investigations are associated with numerous potential problems. These concerns should not be viewed as insurmountable, but it is imperative that they are considered in study design, data analysis and results interpretation [21] (Figure 3). It is especially important to remember that health investigations do not start in the laboratory. They begin in the field. And because surveillance is carried out with the goal of taking action, resources and efforts should not only be allocated to data collection but should fundamentally always include data analysis and dissemination of information.

## Competing interests

The author declares to have no competing interests.
